# Implantation of an Intraosseous Transcutaneous Amputation Prosthesis Restoring Ambulation After Amputation of the Distal Aspect of the Left Tibia in an Arabian Tahr (*Arabitragus jayakari*)

**DOI:** 10.3389/fvets.2019.00182

**Published:** 2019-06-11

**Authors:** Andrzej Golachowski, Masoud Rashid Al Ghabri, Barbara Golachowska, Hamood Al Abri, Marek Lubak, Michal Sujeta

**Affiliations:** ^1^Directorate General of Veterinary Services, Royal Court Affairs, Muscat, Oman; ^2^IWET Veterinary Surgical Implants, Kleosin, Poland

**Keywords:** Intraosseous Transcutaneous Amputation Prosthesis, ITAP, prosthesis, *Arabitragus jayakari*, Arabian Tahr, wildlife, amputation, rehabilitation

## Abstract

**Objective:** This document is to report the clinical application and outcome of custom designed Intraosseous Transcutaneous Amputation Prosthesis (ITAP) in the left hind limb in a wildlife Arabian Tahr (*Arabitragus jayakari*).

**Sample Population:** A 4-year-old, 15 kg Arabian female Tahr from the Omani Mammals Breeding Center.

**Method:** The distal aspect of the left tibia was amputated due to trauma. A custom designed ITAP was inserted into the tibia 5 months after amputation. Two weeks after the surgery an exoprosthesis was attached to the limb. The outcome of the surgery was measured by means of assessments of the limb function and radiographic examination.

**Results:** Fourteen days after surgery the exoprosthesis was attached. The animal was walking showing lameness grade 2/5 (AAEP lameness scale). Within three weeks lameness improved to grade 1/5. Skin integration with the ITAP was achieved within 28 days. The Tahr was successfully reunited with the breeding herd.

**Conclusion:** Application of the ITAP to the left tibia of the Arabian Tahr resulted in positive functional outcomes. Six months post-surgery observations confirmed ambulation with grade 1/5 lameness was restored. The animal was reintroduced to the breeding group.

**Clinical Relevance:** ITAP offers a viable option to restore functionality and ambulation in wildlife.

## Introduction

Although the Arabian Tahr (*Arabitragus jayakari*) is endemic across the mountainous regions of northern Oman and the United Arab Emirates, it is considered an endangered species under the Red List issued by the IUCN (International Union for Conservation of Nature). The Arabian Tahr faces major threats in the form of habitat loss, poaching, and competitive livestock, particularly in the form of goats. The Arabian Tahr population is estimated at fewer than 2,500 individuals and numbers continue to decline despite conservation efforts (1).

In an effort to enhance the scope of work that can be incorporated into conservation efforts, this paper describes the success and implications that were encountered with implanting a custom-made intraosseous transcutaneous amputation prosthesis (ITAP) to the distal aspect of the left tibia in an Arabian Tahr. Most wildlife amputations result from trauma (2). An overload in the contralateral limb may produce tendon and suspensory apparatus breakdown (3). Authors observed that wildlife animals with ambulation problems are the weakest in their group with limited access to food and higher risk of attacks within the group hierarchy. When separated from the herd in safe enclosures, injured Arabian Tahrs develop separation stress, susceptibility to infections and an inability to breed. Compromised animals are often subjected to premature euthanasia (4). Due to significant consequences of limb amputation, all efforts must be made to re-establish movement on four limbs and preserve as much of the limb as possible to allow the application of prosthetic aid. Through elaborating on the surgical technique applied, rehabilitation processes, and encountered complications of implanting an ITAP to restore functionality to the left shin of an Arabian Tahr, this paper seeks to contribute to the existing field of conservation by showcasing reintegration into a breeding herd of Arabian Tahr.

## Clinical Report

### History

A 15 kg, 4-year-old female Arabian Tahr from the Omani Mammals Breeding Center had its left hind leg at the distal tibia amputated 5 months before the prosthesis operation.

The initial injury was a left distal tibia extra-articular multi-fragmented open fracture with severe contamination and soft tissue loss. It was treated by a circular external fixator. Within 5 days ischemic necrosis and infection developed and amputation of the distal tibia was performed.

Post-amputation the animal showed severe problems in ambulation. Different custom-made exoprostheses were tested without positive results. The negative outcomes were caused primarily by attachment failure, stump irritation, and infection. Considering the animal's welfare, an attempt to restore normal movement by the implantation of ITAP was undertaken.

### Prosthesis Design

Preoperative assessment of limb length and diameter of the medullary canal was based on radiographic projections with Kirschner wire that was 5 mm in diameter, 100 and 200 mm in length, as well as photographs of the animal.

CT scanning and 3D image reconstruction of the affected leg were unavailable.

The custom-designed prosthesis was manufactured by the IWET Company in Kleosin, Poland by engineers Marek Lubak and Michal Sujeta.

**Figure 1:**
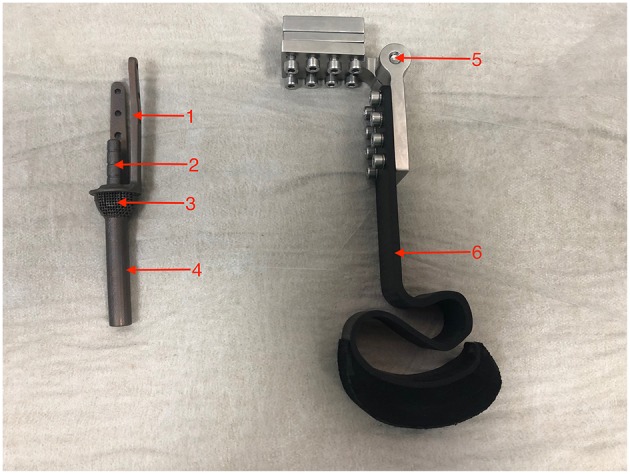
Custom designed prosthesis.

The prostheses elements are pictured in Figure 1. The prostheses are comprised of a titanium alloy (Ti6Al4V) stem for intramedullary placement (2) with dorsal and medial locking plates (1) for additional secure fixation, a perforated umbrella-shaped flange (3) for skin in-growth and a distal extra cutaneous pin (4) for exoprosthesis attachment. The exoprosthesis consisted of an adjustable locking hinge (5) made from stainless steel attached to a carbon fiber leg with the hoof (6). All elements of the designed prosthesis were subjected to structural analysis by finite element methods (FEM). Due to the patient's very active nature, triple weight (45 kg) was assumed for calculations. The titanium prosthesis was made by combining techniques of computer numerical control milling and 3D printing. Elements of the prosthesis were adjustable with the possibility of regulating the length (+/– 40 mm) and a flexion angle for the best fit for the animal.

### Surgical Implantation

The 15 kg Tahr was sedated with 1 mg medetomidine at a dose of 0.06 mg/kg (Domitor 1 mg/ml Orion Pharma) and 1.5 mg butorphanol at a dose of 0.1 mg/kg (Butomidor 10 mg/ml Orion Pharma), followed with 750 mg ceftriaxone at a dose of 50 mg/kg (Rocephin 1 g Roche) and 7.5 mg meloxicam at a dose of 0.5 mg/kg (Meloxicam 5 mg/ml Troy Laboratories) by intravenous injection. After sedation, the leg was prepared for aseptic surgery in the standard manner.

Anesthesia was induced by 30 mg propofol at a dose of 2 mg/kg (Propofol 1% 10 mg/ml Fresenius), then an endotracheal tube that was 5 mm ID, 7.3 mm OD × 20 cm (Mila International) was inserted, cuff inflated, and anesthesia was maintained with 2–5% isoflurane in 100% oxygen.

The patient underwent an electrocardiogram, capnography, pulse oximetry, and indirect blood pressure for monitoring anesthesia. Ringer lactate as a supportive fluid therapy was maintained at a dose of 10 ml/kg/h during the surgical procedure. The patient was placed in the right lateral position with the affected limb facing in the uppermost direction.

**Figure 2:**
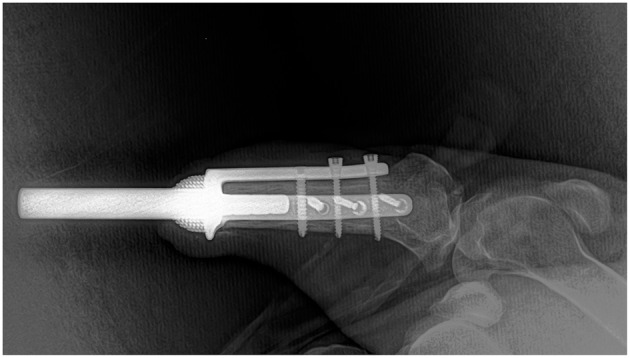
Intraoperative radiograph to assess implant position and alignment.

A circumferential transverse incision of skin was made 1 cm proximal from the stump and extended 4 cm proximally at the dorsal aspect of the tibia. Dissection of the soft tissue from the shaft of the tibia allowed for a 20 mm osteotomy of the distal tibia using an oscillating saw. The medullary canal of the tibia was broached using a curette and a 6 mm drill. The ITAP implant was inserted and pushed into position with a slap-hammer and secured by one 3.5 mm and two 2.7 mm locking screws both at the dorsal and medial plate. Intraoperative radiographs were taken to assess the implant position and its alignment before the final tightening of locking screws (Figure 2).

**Figure 3:**
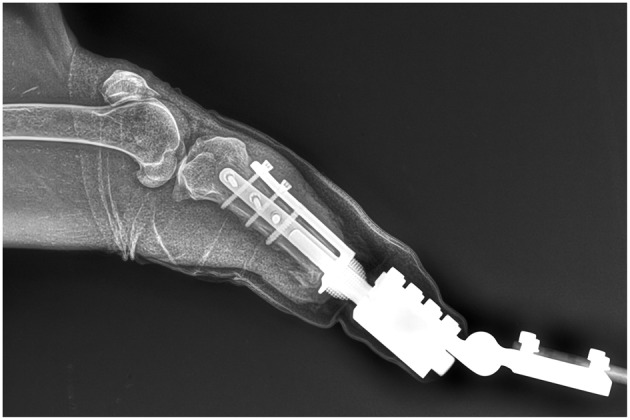
Radiogram at 8 weeks after implantation.

The remaining muscles and tendons were attached to a perforated umbrella-shaped flange with 2-0 polyglactin 910 (VICRYL Plus, Ethicon). The skin was closed with 2-0 polyamide (Ethilon, Ethicon) simple interrupted sutures. Recovery from the anesthesia was uneventful. Postoperative medications included antibiotic therapy with 135 mg combination of amoxicillin, clavulanic acid at a dose of 9 mg/kg (Synulox RTU Zoetis), and pain control with 7.5 mg meloxicam at a dose of 0.5 mg/kg (Meloxicam 5 mg/ml Troy Laboratories) every 24 h for 10 days. The wound dressing was changed every 2 days. The skin seal around the distal flange was achieved after 30 days. A radiographic examination at 8 weeks revealed thickening and bone remodeling around the distal end of the bone (Figure 3).

An exoprosthesis was attached 2 weeks after surgery. After the initial adjustment, including length, and hinge flexion, the animal immediately started bearing its own weight. Initially, lameness was not ideal at a level of 2/5 on the AAEP scale. Within a 3-week period, however, this improved to a 1/5 on the AAEP scale.

**Figure 4:**
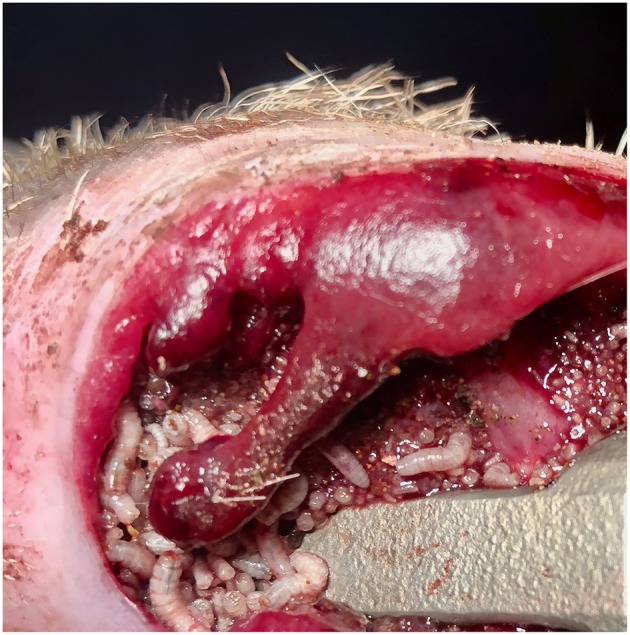
Skin-implant junction infested with larvae of the screwworm fly.

Three months post-implantation a complication occurred. Larvae of the Old-World screwworm fly (*Chrysomya bezziana*), which feeds on living tissue, were found in the junction between the skin and the implant. It was observed that the subsequent infection with sanguine-purulent discharge affected the skin and subcutaneous tissue around the prosthesis (Figure 4). Treatment for the screwworm fly consisted of aggressive tissue debridement, antibiotic therapy, pain management, and wound care. Under general anesthesia, with the same protocol as described previously, surgical debridement, and maggot removal was performed.

Following the removal of the screwworm larvae and maggots, systemic antibiotic treatment with 33 mg of ceftiofur at a dose of 2.2 mg/kg every 24 h (EXCENEL RTU, Zoetis) and 300 mg of amikacin at dose of 20 mg/kg IV every 24 h (Amikin inj 100 mg/ml Bristol-Myers) was administered for 7 days, with a 135 mg combination of amoxicillin and clavulanic acid at a dose of 9 mg/kg (Synulox RTU Zoetis), and pain control with 7.5 mg meloxicam at a dose of 0.5 mg/kg (Meloxicam 5 mg/ml Troy Laboratories) every 24 h for the next 10 days. This combination of medicine administered post-treatment of the infection proved effective.

To promote healing and reduce the risk of implant-bone infection for the first 4 days, interactive moist dressings with the addition of 200 mg amikacine (Tenderwet active 24, Hartmann) combined with silver mesh (Atrauman Ag, Hartmann) were applied and changed daily. After 4 days, the remaining skin defect was treated with hydrocolloid dressing (Hydrocoll, Hartmann) combined with silver mesh (Atrauman Ag, Hartmann) and wound healing cream, containing *Sh-Polypeptide-1-* acidic Fibroblast Growth Factor (Regen Gel, VET STEM CELL Poland), applied daily. The skin defect completely healed in 18 days. Radiography examination revealed bone remodeling around the distal end of the tibia without any signs of osteolysis. During the treatment, the animal was kept indoors for close observation. Post-treatment the Tahr was released from a safe enclosure into its herd.

The distal part of the tibia was constantly protected with soft dressing (Softban) and secured by an adhesive bandage (Tensoplast) all the time and changed every 7–10 days. The patient tolerated the situation very well; the dressing was changed without sedation and no more skin-implant complications have been observed.

With time, it was found that an S-shape design of the “hoof” in the prosthesis is not optimal, as it hooks on long grass or even might cause leg entrapment with wire mesh fences. The gap on the distal end of the prosthesis was closed by silver duct tape (Scotch).

After 6 months post-surgery, the animal was successfully returned to the herd, where it was accepted and now walks and runs using the prosthetic. Although a shortening of stride was noticeable throughout the rehabilitation process, the restoration of lameness from 2/5 to 1/5 (on the AAEP scale) within 3 weeks was a positive indicator of functional restoration success.

## Discussion

The two kinds of prosthesis devices that are used in veterinary practice are socket-based prostheses and intraosseous transcutaneous amputation prostheses (ITAP). Exoprostheses, or socket-based prostheses, are designed for patients with partial amputations that still have fully functional remaining structures of the shoulder and elbow or the hip and stifle. The prosthesis is fixed to the animal by a system of ropes or Velcro and, depending on the fitting and comfort of the patient's prosthesis, it can be worn all day or for shorter periods of time such as in cases of brief walks (5). Maintaining an exoprosthesis in wildlife is difficult because of the everyday challenge of checking the cleanliness or adjusting the exoprosthesis.

Intraosseous transcutaneous amputation prostheses (ITAP) have been investigated as an option for patients since the 1990s (7). A prosthesis is attached to the bone by a titanium implant inserted in the marrow cavity which protrudes through the skin. This eliminates complications of socket prosthesis arising from stump-prosthesis interactions, thereby resulting in skin irritation, pressure sores, discomfort, and a limited range of motion (8). Both exoprosthesis and ITAP are time-consuming and stressful for the animal. One case report, however, describes the successful use of a custom-made exoprosthesis in a free-range red deer during a 6-month hospitalization period (6). The current reported use of ITAP in veterinary clinical cases are limited but provide promising results whilst also describing complications observed during these pioneering surgeries.

A study conducted over a period of 24 months on 8 skeletally mature 18–24 month-old sheep, for instance, showed implant failure in one case, tendon breakdown in another sheep, whilst the remaining 6 sheep completed a 24-month observation period successfully (9). Similar success was documented from the first reported case of a dog with a bilateral pelvic limb amputation receiving custom-made titanium implants surgically attached in both hind limbs due to trauma. The case reported that the dog started to ambulate with assistance immediately after surgery. Seven days post-surgery the dog demonstrated the ability to walk without assistance. The dog's locomotion functions were restored, allowing it to walk, trot and run (10). In a parallel case, four dogs received ITAP after amputation of the distal aspect of the limbs due to malignant neoplasia dermal integration with titanium. Prostheses were achieved within 3 weeks and they were walking using their prosthetic limbs within 8 weeks (11). In contrast to these successes, the implantation of a partial limb prosthesis in a White-Naped crane (Grus vipio) resulted in euthanasia within 3 weeks due to vascular compromise on the opposite leg. The leg became progressively devitalized (12, 13). The decision to use this type of prosthesis was based on previously published results of the use of ITAP in ovine experimental models, clinical cases in dogs and the failure of other therapies.

Applying the ITAP to the left tibia of the Arabian Tahr resulted in positive functional outcomes. Six months post-surgery ambulation with grade 1/5 lameness was restored and the animal was reintroduced to the breeding group. The decision to ITAP prosthesis was based on previously published results of the use in ovine experimental models, clinical cases in dogs and the failure of other therapies.

The literature on ITAP surgeries describes high complication rates after amputation in wildlife such as wapiti, domesticated sheep, and goats. From 13 wapiti that had to undergo limb amputation, 6 were euthanized due to postsurgical complications (2). High levels of complications were also found in goats and sheep. Retrospective analysis revealed that 7 out of 15 goats and 2 out of 7 sheep underwent amputation and in post-surgery, it was observed that they experienced uncoordinated gait, tendon breakdown, laxity of the contralateral limb, chronic lameness, surgical site infection, chronic intermittent pain, and angular limb deformity (2). Compromised animals were euthanized (5).

Given the significant consequences of limb amputation, all efforts must be made to re-establish movement on four limbs and preserve as much of the limb as possible to allow for successfully applied prosthetic aid. Exoprostheses, or socket-based prostheses, are designed for patients with partial amputations that still have fully functional remaining structures. The stump of the exopostheses requires daily cleaning and regular maintenance. The complication of wearing an exoprosthesis arises from a poor fit resulting in pain, skin infection, irritation, ulceration, and pressure necrosis. Most amputees will experience such complications at some point, the reason being that the load bearing forces are transmitted by the prosthesis to the stump skin, which is not suitable to receive such forces (3). When such problems arise, the patient's quality of life is limited and they are unable to wear a prosthesis until the complications are resolved.

In the presented case study, the Tahr spent most of its time in recumbency and exhibited severe problems balancing by frequently falling to the left side when walking after amputation. Different custom-made socket prostheses did not work because they would become loose or detached within 6–24 h, resulting in stump skin damage, irritation, and infection. The prosthesis procedure had to be completed as soon as possible before collateral support limb breakdown, muscle loss, and further deterioration of the condition of the Tahr could take place.

The animal's inherently calm character made it agreeable to work with when conducting procedures like injections and wound dressing changes. This proved to be an indisputable advantage during the post-operation and rehabilitation period. In the collective opinion of the authors, the aforementioned factors should always be considered when screening the applicability of ITAP procedures in wildlife patients.

ITAP prosthesis is at risk of infection both within the junctions between the skin and the implant as well as in the bone implants themselves. Most prevalent pathogens are strains of Staphylococcus sp. which affect 18–30% of patients. Topical and systemic use of antibiotics for a prolonged time does not always reduce infection rates and can sometimes result in multi-drug resistant bacterial strains with biofilm formation (14).

The surgical procedure of ITAP in human patients consists of two different surgeries in order to significantly reduce the risk of implant infection. The first surgery involves debulking the soft tissue and implanting the endo-module into the medullary canal by a tight-press fit. After 6 weeks the second surgery attaches a skin-perforating connector to the endo-module (15). The described surgical procedures in experimental ovine models and canine clinical cases consist of one-stage intramedullary implantation of a device by a tight-press fit method. This method works well in most cases but may result in complications due to an insecure fit of the implant in the medullary canal as observed in an ovine model (15, 16).

Despite the higher infection rate associated with implants with brackets compared to implants without brackets in human patients (15), it was decided to use titanium brackets in the design of the implants for the Arabian Tahr to secure the implant with locking screws. This decision was made taking into account the fact that the Tahr is naturally very active. The brackets used were smooth and made from titanium alloy (Ti6Al4V), each secured with one 3.5 mm and two 2.7 mm locking screws to provide extra stabilization during the initial period of osteointegration. At the time of writing the article, 6 months post-implantation, there are no signs of infection nor other adverse effects due to the brackets. In our opinion the brackets have helped to stabilize the implant and allow for early mobilization at 14 days after surgery.

The post-operative rehabilitation program designed for the Arabian Tahr was different from the ones described by other authors. Unlike the experimental protocol followed on an ovine amputation model which allowed animals to walk free on prosthesis immediately after surgery (7), the expoprosthesis was attached to the described case of the Arabian Tahr 2 weeks after surgery once the skin wound had healed. Immediately after securing the exoprosthesis to the Tahr, it showed the ability to bear weight and walk. This is a shorter wait period than what was described in the case outlined by Fitzpatrick, where a waiting period of 5–6 weeks was employed before attaching exoprosthesis, and the implant-stump area was protected by an external fixation apparatus working as a walking frame (11).

A study conducted on 9 sheep for 12 months demonstrated that 4 weeks post-surgery the sheep were able to load the prosthetic limb with nearly 80% of their pre-amputation loading condition. After 1 year, however, the load had dropped to 74%. None of the animals returned to full weight-bearing on the prosthetic limb, but at the same time, a symmetric gait was observed (10).

The restoration of mobility and locomotive functionality in the case of the injured Arabian Tahr supports the use of adjustable constructs and fixtures when it is not possible to make perfect measurements due to inaccessibility to advanced imaging such as CT. Exoprosthesis was attached 14 days after surgery using hinges that were adjustable and had a locking ability. Using these proved effective in fitting the prosthesis perfectly and in a manner that accommodated the animal's needs. It was also observed that the Tahr held a symmetric gait with a lameness grade of 2/5 (on the AAEP lameness scale). Within 3 weeks, however, lameness had improved to a grade of 1/5. Lameness has remained 1/5 on the AAEP scale for the duration of the observation period thus far.

It was found that the skin-implant interfaces are susceptible not only to infection but also to parasitic invasions like the larvae of the Old-World screwworm fly. The risk of maggot invasion is associated with secondary infection, and inflammation could result in fatal complications as the larvae feed on live tissue causing both toxicosis and secondary septicemia. Only early diagnosis and aggressive treatment of both surgical tissue debridement and antibiotic therapy prevented implant failure. Complications like these should be considered, particularly when dealing with patients that live in affected areas. To minimize the risk of such infections and the consequences associated with it, some kind of barrier-like soft wound dressing must be applied at all times.

**Figure 5:**
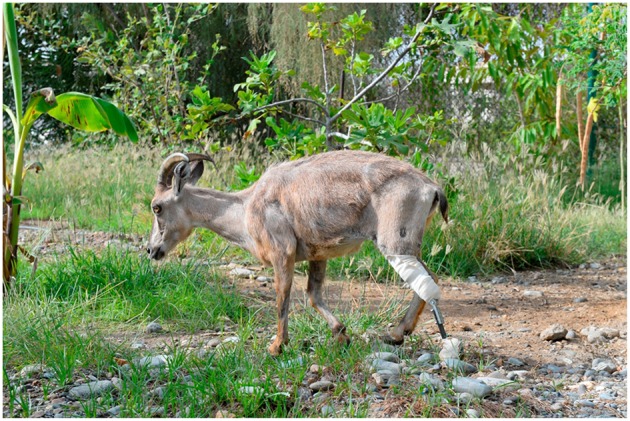
Arabian Tahr 6 months after prosthesis implantation.

At the time of designing the exoprosthesis the safety of the animal should always be considered. Prosthesis for free-ranging animals cannot consist of elements that might hook on objects. Observations conducted for 6 months after surgery confirmed that the main goal of the treatment, which was restoring ambulation in a pain-free manner and reintroducing the Tahr into the breeding group for conservation purposes of endangered species, was achieved (Figure 5). Further, close monitoring might give some valuable information about the long-term outcome of ITAP in wildlife.

## Ethics Statement

The animal is under care of Omani Mammals Breeding Center, which is a part of Directorate General of Veterinary Services RCA in Oman. An oral approval for surgery was given by Director General Eng Mahmood al Abri.

## Author Contributions

All authors contributed equally to the study design, preparation and final approval of the manuscript. AG presented idea, performed surgery and supervised aftercare and rehabilitation process, prepared and corrected the manuscript. MA, BG, and HA assisted with surgery, were involved with aftercare and rehabilitation process, prepared and corrected the manuscript. ML and MS designed and manufactured prosthesis, prepare and correct manuscript.

### Conflict of Interest Statement

The authors declare that the research was conducted in the absence of any commercial or financial relationships that could be construed as a potential conflict of interest.
